# Litholapaxy

**Published:** 1880-04-20

**Authors:** A. F. Sanger

**Affiliations:** Bangor


### *A CASE OF LITHOLAPAXY.*

BY A. F. SANGER, M.D., BANGOR.

During the fall of 1879 I was called to Mr. B. N. T., aged sixty-four, with a catarrhal, asthmatic, and lymphatic diathesis. He had to give up business in May, 1877, and had been confined to his bed since January, 1879, with straining and constant desire to pass water every ten to thirty minutes, pain on change of position, and urine heavily loaded with pus and phosphates; pulse ranging from 90 to 100. Sound 22, French, detected a stone apparently at the base of the bladder, the impression being conveyed to the hand that the heel of the sound impinged on the stone.

He had lost sixty-five pounds of flesh. His age, condition, and the apparent magnitude of the stone augured ill for lithotomy or ordinary lithotrity. Litholapaxy, or the rapid lithotrity, seemed the only solution of my dilemna. By advice of Dr. Bigelow, I sent to New York for his evacuating apparatus and lithotrite.

Knowing Sir Henry Thompson's aversion to prolonged crushing, his want of confidence in the tolerance of the bladder, his opposition to evacuating tubes above 25 French, with some hesitation and delay I proceeded with the operation. Another discouraging feature was that the patient took ether poorly, the throat getting constantly clogged with mucus, and the pulse running up to 120, and after three-fourths of an hour of etherization to 140 and 160. The above symptoms prevented us from testing the full tolerance of the bladder and a successful completion of the operation at a less number of sittings. It was therefore with some misgivings that I used the Bigelow lithotrite and the No. 29 French curved tube.

The operation proved full of difficulties. Forty minutes of continued search disclosed the stone back of or above the symphysis pubis, apparently fastened in a way that only the point of the sound or lithotrite could scrape over a fixed mass. I nipped away and evacuated thirty grains in the next twenty minutes. Atfer this, at short intervals, there were two operations, the first lasting seventy minutes and evacuating ninety grains of stone; the second, one hundred and five minutes, with a product of one hundred and twenty grains. In both instances searching and crushing occupied two-thirds of the time, and the handle of the lithotrite had to be carried back between the legs as far as it would go. At the last operation I succeeded in grasping a stone two inches in diameter, but not in detaching and breaking it up, as the lithotrite seemed to plow through it. As the patient had become very much exhausted. I evacuated rapidly and left some loose pieces in the bladder. I drew out one piece engaged in the eye of the tube, too big to come through the tube. The detritus occasioned high fever and cystitis, so that nothing more was done for three or four weeks. On one of these occasions I tried Ferguson's fenestrated lithotrite without engaging the stone, but several times seizing the mucous membrane, which was never done with the Bigelow instrument.

Between the third and fourth operations, I met Dr. Bigelow in Boston, who expressed the belief that, the stone being large, and lying across the floor of the bladder, the beak had passed under the center of it.

December 16th. I etherized the patient again, elevated the hips, and by forcibly depressing the handle of the lithotrite tilted it up, and seized, crushed and evacuated nine hundred and thirty grains of stone in seventy minutes. This time the lithotrite was introduced three times, occupying about half of the time. Very many pieces larger than peas were removed. Not daring to keep the patient any longer under ether, I left two or three pieces which I could hear click against the tube. December 22d. Crushed again, and evacuated in forty minutes sixty grains, one piece weighing twenty grains being extracted in the eye of the tube.

Thus in five and three quarters hours at five different operations twelve hundred grains were extracted, and at each time the patient suffered no detriment from the prolonged continuance of the instruments in the bladder, excepting the time when a considerable number of loose pieces were left unevacuated. I should have used the 31 French straight tube, had not my greatest difficulty been in engaging the stone between the jaws of the instrument. The patient was on his feet in about ten days, and could hold water nearly all night. I have written a rather lengthy detail of the case because I consider it a most complete triumph of Bigelow's instruments under the most trying circumstances.—*Boston Med. and Surg. Journal.*

